# Synthesis and Antiangiogenic Activity of Novel Gambogic Acid Derivatives 

**DOI:** 10.3390/molecules17066249

**Published:** 2012-05-25

**Authors:** Tao Chen, Rong-Hong Zhang, Shi-Chao He, Qin-Yuan Xu, Liang Ma, Guang-Cheng Wang, Neng Qiu, Fei Peng, Jin-Ying Chen, Jing-Xiang Qiu, Ai-Hua Peng, Li-Juan Chen

**Affiliations:** 1 State Key Laboratory of Biotherapy, West China Hospital, West China Medical School, Sichuan University, Chengdu, Sichuan 610041, China; 2 School of Chemical Engineering, Sichuan University, Chengdu, Sichuan 610065, China

**Keywords:** gambogic acid, antitumor, antiangiogenesis, zebrafish, toxicity

## Abstract

Gambogic acid (GA) is in a phase II clinical trial as an antitumor and antiangiogenesis agent. In this study, 36 GA derivatives were synthesized and screened in a zebrafish model to evaluate their antiangiogenic activity and toxicity. Derivatives **4**, **32**, **35**,**36** effectively suppressed the formation of newly grown blood vessels and showed lower toxicities than GA as evaluated by zebrafish heart rates and mortalities. They also exhibited more potent migration and HUVEC tube formation inhibiting activities than GA. Among them, **36** was the most potent one, suggesting that it may serve as a potential new antiangiogenesis candidate with low toxicity. Additionally, **36** showed comparable antiproliferative activity to HUVECs and five tumor cell lines but low cytotoxicity to LO2 cells.

## 1. Introduction

Angiogenesis, or new blood vessel growth, is defined as a process in which a network of new blood vessels emerge from preexisting vessels. The inhibition of angiogenesis is a promising tool to fight cancer [1]. Tumor angiogenesis, which involves multiple cellular processes including endothelial cell (EC) activation, invasion, migration, proliferation, tube formation and capillary network formation, is essential for cancer growth and metastasis [1,2,3,4]. Without the development and progression of new blood vessels, tumors can rarely grow over a few mm^3^ in size or metastasize to other organs [5,6]. Therefore, inhibition of angiogenesis is an important target for cancer therapy [7,8].

The zebrafish (*Danio rerio*) screening model for anti-angiogenesis activity has become increasingly popular in different fields such as pharmacology, toxicology and developmental and evolutionary biology since the 1970s [9,10,11]. It is regarded as a powerful tool in the study of human diseases [9]. Indeed, zebrafish possess many advantages that make them a highly useful valid tumor model system for antiangiogenesis, including relatively small size (up to 3 cm), low cost, and being easy to house and maintain in large quantities [9,10]. The application of transgenesis to the zebrafish resulted in the ability to spatially and temporally control gene expression and to create or increase *in vivo* imaging capabilities [9]. Transgenic zebrafish models expressing green fluorescent protein (GFP) in vascular endothelial cells (ECs) are significantly useful for studying the formation of the vasculature *in vivo* [12].

Cultured ECs are also widely appreciated tools in angiogenesis research [5]. ECs commonly used for more than 30 years now are human umbilical vein ECs (HUVECs) which are easily obtained and less prone to acquire drug resistance [11]. EC migration *in vivo* is indispensable for wound-healing as well as for the formation of new vessels [4]. The capacity of the ECs to form tube-like structures when cultured on a semi-natural matrix is another typical *in vitro* angiogenesis assay [2]. Therefore, the phenotype of tumor ECs is subject to investigation to identify putative target molecules for interference to discover novel angiogenesis-inhibiting agents.

Gambogic acid is a natural product isolated from the resin of the *Garcinia hurburyi* tree found in Southeast Asia. It is in a phase II clinical trial in China and is widely known as an antiangiogenesis and antitumor agent [13,14,15,16,17,18,19]. However, until now, no study has focused on the structural modification of GA to evaluate antiangiogenic activity and related toxicity. In this study, the antiangiogenic activities and toxicities of GA and GA derivatives were evaluated for the first time using a zebrafish screening model.

In this paper, we synthesized and screened 36 GA derivatives. Compounds **4**, **32**, **35**, **36** effectively suppressed the formation of newly grown segmental blood vessels in the zebrafish-based assay and were less toxic to zebrafish compared to GA. These four derivatives also exhibited more potent inhibitory potencies against the migration and tube formation of HUVECs *in vitro* than GA. Importantly, among them, **36** was the most potent one, with a 98.3% migration inhibition rate and 100% tube formation inhibitory rate at a concentration of 2 μM, suggesting that this derivative may serve as a potential new antiangiogenesis candidate.

## 2. Results and Discussion

### 2.1. Chemistry

Gambogic acid was isolated from the easily and widely available gamboge resin in an overall yield of approximately 5%. It was purified by changing the crude extract from the gamboge resin into a pyridine salt, followed by recrystallization [20]. There are many functional groups in the structure of GA which could potentially be modified such as 30-carboxy, 8-ketone, methyl group at C-35 or C-39, 6-hydroxy, 9,10-double bond in the α,β-unsaturated ketone, carbon–carbon double bond at C-32/33 or C-37/38. However, earlier structure activity relationship (SAR) studies had identified that the 9,10-double bond in the α,β-unsaturated ketone is critical for activity, and the modifications of 6-hydroxy and 8-ketone did not improve the activity dramatically [21,22]. Therefore, to develop novel GA derivatives as inhibitors of angiogenesis, we elected to modify the 30-carboxy and carbon–carbon double bond at C-32/33 and C-37/38.

**Scheme 1:**
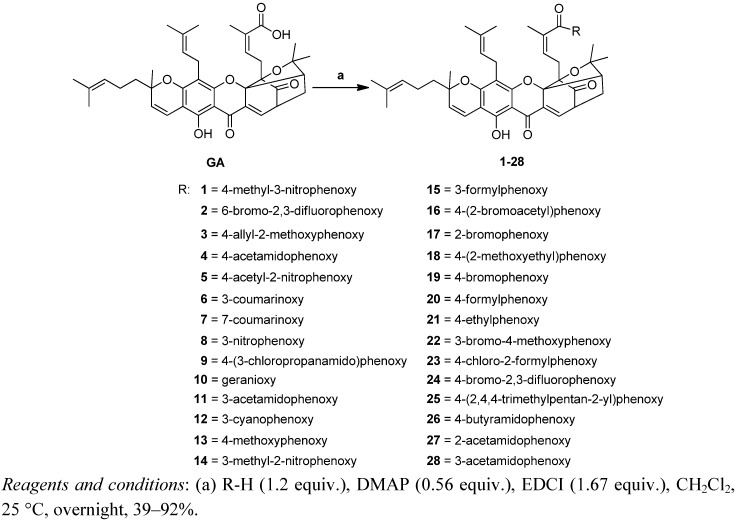
Synthesis of derivatives **1–28**.

**Scheme 2:**
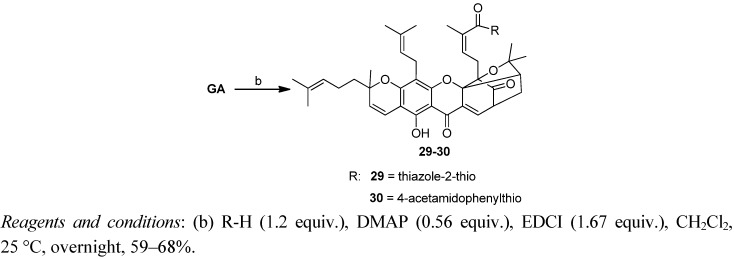
Synthesis of derivatives **29–30**.

**Scheme 3:**
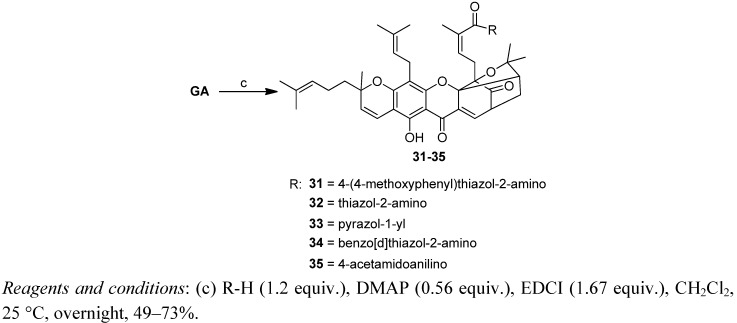
Synthesis of derivatives **31–35**.

The modification of the 30-carboxy group is depicted in Scheme 1, Scheme 2 and Scheme 3. Coupling of GA with various alcohols or phenols in the presence of DMAP and EDCI produced the corresponding GA esters **1–28** in 39%–92% yield (Scheme 1). Reaction of GA with two sulfhydryl compounds in the presence of DMAP and EDCI produced the corresponding GA thioesters **29–30** in 59%–68% yield (Scheme 2). 

Reaction of GA with appropriate amines in the presence of DMAP and EDCI produced the corresponding amides of GA **31–35** in 49%–73% yield (Scheme 3).

**Scheme 4:**
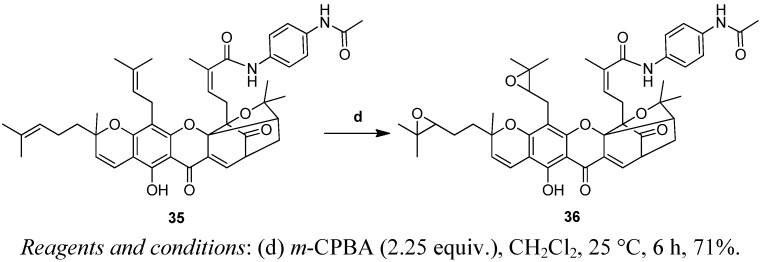
Synthesis of analogue **36**.

The carbon-carbon double bonds at C-32/33 and C-37/38 were modified as shown in Scheme 4 [21]. Reaction of **35** with *m*-CPBA for about 6 h at 25 °C, produced the GA derivative **36** containing two epoxy groups at C-32/33 and C-37/38 in 71% yield.

### 2.2. Antiangiogenic Activity and Toxicity in the Transgenic Zebrafish Model

As stated previously, zebrafish is regarded as a powerful tool in the study of human diseases. Transgenic zebrafish models expressing green fluorescent protein (GFP) in vascular endothelial cells (ECs) are significantly useful for studying the formation of the vasculature *in vivo*. In this study, all GA derivatives were incubated with the transgenic friend leukemia integration-1 (*fli-1*): enhanced GFP zebrafish embryos which carried a *fli-1* promoter which can drive the expression of GFP in the whole endothelium [23].

At a concentration of 1 μM, compounds **16**, **20**, **22**, **23** and **31** were inactive, the antiangiogenic rates of compound **9** was less than 25%, and the antiangiogenic rates of compounds **4**, **26**, **32**, **35**, **36** and GA were 25%–50% (Table 1). The remaining derivatives (not listed) were inactive even at concentrations exceeding 10 μM. However, GA caused zebrafish embryo death at concentrations of 2.5 μM and 10 μM. The antiangiogenic activities of **9** and **26** were moderate at 2.5 μM and not enhanced greatly when the concentration reached 10 μM. 

**Table 1:** Antiangiogenic effects in zebrafish embryos.

Compounds	Anti-angiogenic phenotype ^a^		
	1 μM	2.5 μM	10 μM
**4**	**++**	**+++**	**++++**
**9**	**+**	**++**	**++**
**16**	О	О	**++**
**20**	О	О	**++**
**22**	О	О	**++**
**23**	О	О	**++**
**26**	**++**	**++**	**++**
**31**	О	**+**	**++**
**32**	**++**	**+++**	**++++**
**35**	**++**	**+++**	**++++**
**36**	**++**	**+++**	**++++**
**GA**	**++**	**Dead ^b^**	**Dead**

^a^ The semiquantitative scale for angiogenic inhibitory rates, each group comprised 10 fishes, this experiment was repeated three times: О, inactive; ++++, >75% angiogenic inhibition; +++, 50–75%; ++, 25–50%; +, <25% suppression of angiogenesis as compared to the vehicle-treated (0.05% DMSO) zebrafish embryos. ^b^ >50% zebrafish embryos were dead when treated with the indicated concentration of compound.

**Figure 1:**
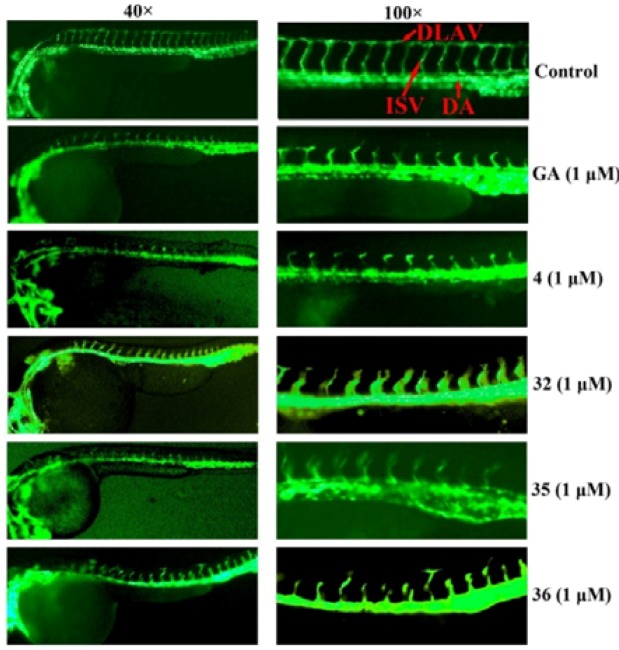
Effects on neovascularization in zebrafish embryos. The transgenic *fli-1*: enhanced GFP zebrafish embryos in embryo water (0.2 g/L of instant ocean salt in distilled water) incubated with 1 μM of compounds at 6 h postfertilization (hpf) for 24 h. Zebrafish embryos were imaged (Imager.Z1, magnification: left, 40×; right, 100×).

**Figure 2:**
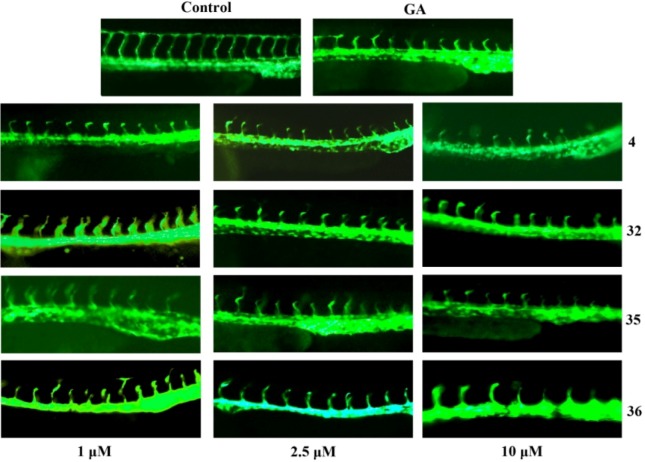
Effects of different concentrations of **4**, **32**, **35**, **36** on neovascularization in zebrafish embryos (Imager.Z1, magnification: 100×).

Importantly, derivatives **4**, **32**, **35**, **36** exhibited strong antiangiogenic activities inhibiting intersomitic blood vessels from the dorsal aorta of zebrafish in a concentration-dependent manner (Figure 1 and Figure 2).

To investigate the toxicity of these four compounds, the heart rates and the mortality rates of zebrafish embryos were recorded (Table 2 and Table 3). The heart rate of zebrafish in the control group was 117 beats/min. At a concentration of 1 µM, treated with compounds **4**, **32**, **35**, **36** respectively, the heart rates of zebrafish were slightly reduced to about 80 beats/min but the heart rate of zebrafish treated with GA decreased to 36 beats/min. Moreover, at 2.5 µM and 10 µM GA, 73.3% and 100% of zebrafish embryos, respectively, were dead, whilst when treated with each of the four compounds at the same concentrations, the mortality rates were less than 20% and the heart rates were still about half of the control group. Therefore, we suggest that compounds **4**, **32**, **35**, **36** were less toxic than GA in zebrafish screening model.

**Table 2:** The effects of **4**, **32**, **35**, **36** on heart rates of zebrafish.

Compounds	Heart rates of zebrafish(beats/min) ^a^		
	1 μM	2.5 μM	10 μM
**4**	84 ± 3.22 ***	80 ± 5.31	54 ± 4.00
**32**	72 ± 6.13 **	60 ± 4.36	54 ± 3.68
**35**	87 ± 8.99 **	57 ± 2.61	45 ± 4.95
**36**	84 ± 3.24 ***	60 ± 5.2	60 ± 2.86
**GA**	36 ± 3.32	**Dead** **^b^**	**Dead**
**Control**	117		

^a^ Data are mean of three experiments (each experiment comprised 10 fishes). ^b^ >50% zebrafish embryos were dead when treated with the indicated concentration of compound. ** *p* < 0.01, *** *p* < 0.005 compared with GA.

**Table 3:** The effects of **4**, **32**, **35**, **36 **on the zebrafish mortality rate.

Compounds	Mortality rates (%) ^a^		
	1 μM	2.5 μM	10 μM
**4**	0	6.7 ± 5.77 **	20 ± 0.00 ***
**32**	0	6.7 ± 5.77 **	16.7 ± 5.77 ***
**35**	3.3 ± 5.77 *	10 ± 0.00 ***	16.7 ± 5.77 ***
**36**	0	3.3 ± 5.77**	16.7 ± 5.77 ***
**A**	20 ± 0.00	73.3 ± 5.77	100
**Control**	0		

^a^ Data are mean of three experiments (each experiment comprised 10 fishes). * *p* < 0.05, ** *p* < 0.01, *** *p* < 0.005 compared with GA.

### 2.3. Effects on the HUVECs Migration

**Figure 3:**
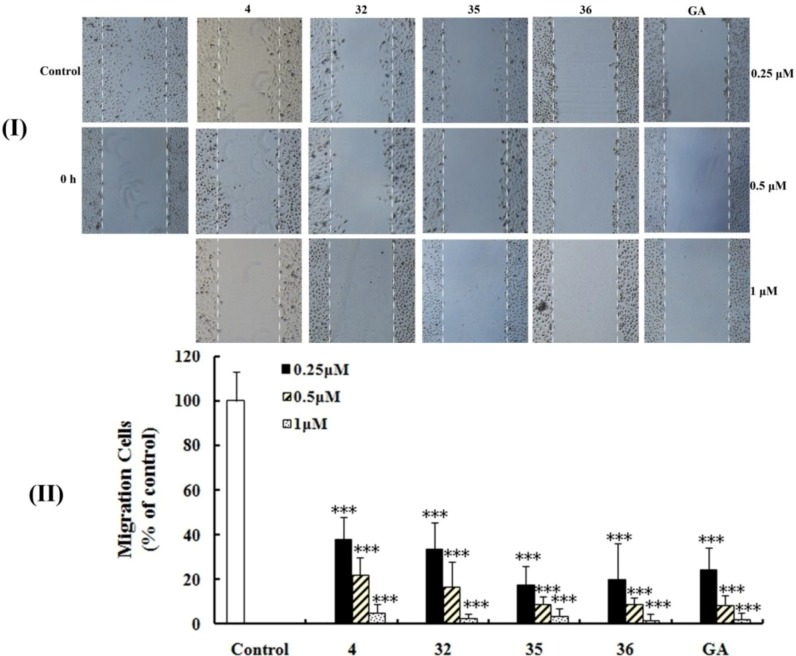
Effects on the HUVECs migration. (**I**) HUVECs suspended in serum-free Dulbecco’s Modified Eagle Medium (DMEM) containing compounds (0.25 or 0.5 or 1 μM) for 24 h were photographed under a phase contrast microscopy (Olympus CKX41, magnification: 50×). Control was treated with serum-free DMEM which was consided as resulting in 100%; (**II**) Migration rates of compounds on the HUVECs. Data represented the mean standard deviation (SD) from three independent experiments. *** *p* < 0.005compared with control.

The migration of ECs is an important step of angiogenesis [16,24]. The continuity of vascular ECs is the most important factor in preserving vessel wall function. Migration of ECs is essential to the recovery of this continuity after vascular ECs injury [24]. Since compounds **4**, **32**, **35 **and **36** presented more antiangiogenic potency than GA, a would-healing migration assay was therefore applied to evaluate their ability to inhibit HUVECs migration [8]. As illustrated in Figure 3(I), the HUVECs migrated into the wound area actively (between the two white dash lines) under the compound-free condition (control). 

At a concentration of 0.25 μM, these four compounds showed significant differences compared with control, but only compounds **35**, **36** manifested more potency compared with GA, and stronger inhibition than those of **4** and **32**. At a concentration of 0.5 μM, derivatives **35**, **36** had similar potency to GA. At a concentration of 1 μM, compounds **35**, **36** were nearly equal to GA and the migratory rates were 3.6, 1.7 and 2.2% respectively [Figure 3(II)]. The effects of these compounds were not due to cell death, because the HUVECs did not show any morphological changes include blebbing, cell shrinkage, nuclear fragmentation, chromatin condensation and chromosomal DNA fragmentation.

### 2.4. Effects on the HUVECs Tube Formation

In the later stages of angiogenesis, tube formation of ECs is also an important process [16]. Thus, a Matrigel tube formation assay was applied to assess the ability of HUVECs to form ECs vascular structures which is believed to be significant in new vessel formation [24].

**Figure 4:**
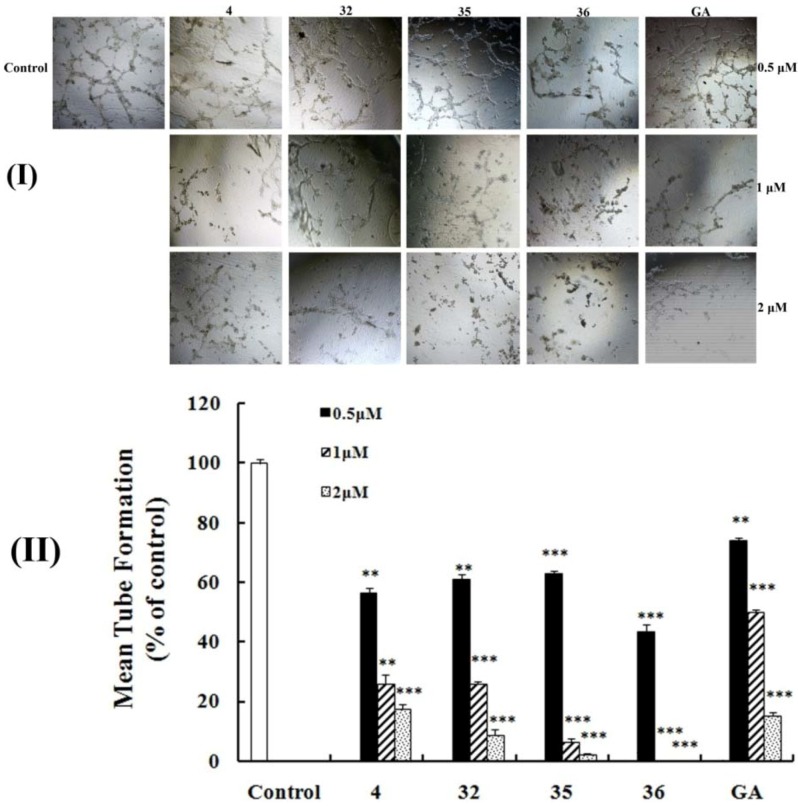
Effects on the HUVECs tube formation. (**I**) HUVECs (1 × 10^4^ cells) suspended in DMEM containing each compound (0.5 or 1 or 2 μM) were added to the Matrigel. Control was treated with DMEM alone. After incubation for 6 h at 37 °C, capillary networks were photographed and quantified (Olympus CKX41, magnification: 100×); (**II**) Rates of the HUVECs tube formation. The number of intact tubes was counted in five randomly chosen regions and expressed as the percentage of the control. The results were expressed as mean ±SD. ** *p* < 0.01; *** *p* < 0.005 compared with control.

In the control, HUVECs showed the highest mobility on Matrigel and formed an intact tubular network in 6 h (Figure 4I). In comparison with the control group, the inhibitory rates of samples treated with **4**, **32**, **35**, **36** and GA at a concentration of 0.5 μM were 43.5, 39.1, 37.0, 56.5 and 26.1%, respectively. Among them, **36** had the strongest inhibitory activity with a 2-fold improvement in the inhibition of tube formation when compared with GA. Most significantly, at concentrations exceeding 1 μM, derivative **36** could completely inhibit tubular structures (Figure 4II), demonstrating that **36** was the most potent antiangiogenesis agent.

### 2.5. Antiproliferative and Anticancer Activity

Inhibiting the proliferation of ECs is another important strategy for antiangiogenesis [25]. Therefore, this experiment was designed to demonstrate the effects of the compounds on antiangiogenesis, HUVEC migration and tube formation. As the exposure time of these derivatives was 24 h in the migration assay and 6 h in the tube formation assay, the effect of the incubation for 24 h in the presence of these compounds on HUVECs proliferation was then tested. Cell viability was assessed by the MTT method [25]. We can see from Table 4 that these compounds had different antiproliferative activities. 

**Table 4:** Inhibition of cell proliferation by compounds **1–36** and GA.

Compound	Cell lines (IC50, μM) ^a^						
	A549	HepG2	HCT116	K562	HeLa	HUVEC	LO2
**1**	43.50 ± 3.92	30.83 ± 2.76	25.53 ± 1.79	10.25 ± 0.48	32.89 ± 1.69	45.20 ± 2.64	ND
**2**	49.15 ± 5.16	41.43 ± 3.52	45.27 ± 4.20	16.10 ± 1.74	57.00 ± 0.62	31.90 ± 1.27	ND
**3**	38.03 ± 3.10	28.83 ± 2.84	18.68 ± 0.97	8.18 ± 1.22	13.07 ± 0.77	52.67 ± 1.15	ND
**4**	0.95 ± 0.09	1.10 ± 0.06	0.48 ± 0.04	0.41 ± 0.02 *	1.34 ± 0.04	1.17 ± 0.12	0.83 ± 0.03
**5**	5.53 ± 0.40	9.45 ± 1.42	5.47 ± 0.24	2.03 ± 0.20	6.37 ± 0.11	4.39 ± 0.27	4.03 ± 0.34 ^##^
**6**	19.70 ± 2.62	25.77 ± 1.76	14.53 ± 1.22	5.47 ± 0.15	33.30 ± 1.83	14.60 ± 0.34	>20
**7**	15.43 ± 2.57	10.77 ± 1.10	6.00 ± 0.40	2.50 ± 0.12	16.10 ± 0.55	10.90 ± 0.72	8.28 ± 0.33 ^###^
**8**	>80	>80	66.40 ± 1.10	17.95 ± 1.38	>80	77.50 ± 3.65	ND
**9**	3.75 ± 0.35	5.16 ± 0.55	2.05 ± 0.41	1.14 ± 0.03	3.42 ± 0.19	4.27 ± 0.25	1.00 ± 0.10
**10**	>80	>80	>80	>80	>80	>80	ND
**11**	3.31 ± 0.45	3.64 ± 0.53	3.13 ± 0.30	2.09 ± 0.19	3.31 ± 0.24	3.48 ± 0.18	3.94 ± 0.19 ^###^
**12**	10.70 ± 0.26	7.80 ± 0.11	5.57 ± 0.26	1.79 ± 0.08	7.10 ± 1.43	7.58 ± 1.36	5.15 ± 1.19 ^###^
**13**	66.75 ± 4.59	60.50 ± 4.24	17.47 ± 1.59	8.05 ± 0.28	>80	27.67 ± 2.77	ND
**14**	>80	40.63 ± 2.75	34.80 ± 1.84	22.60 ± 0.53	>80	41.95 ± 2.76	70.83 ± 2.47 ^###^
**15**	32.95 ± 1.50	10.85 ± 0.06	6.80 ± 0.78	6.11 ± 0.39	62.57 ± 3.12	13.64 ± 1.16	14.83 ± 1.19 ^###^
**16**	>80	4.51 ± 0.29	5.08 ± 0.77	3.46 ± 0.34	8.98 ± 0.58	11.93 ± 1.36	5.25 ± 0.51 ^###^
**17**	>80	61.90 ± 2.69	33.00 ± 1.27	27.00 ± 1.41	>80	29.90 ± 1.44	72.83 ± 2.84 ^###^
**18**	66.15 ± 1.65	6.45 ± 0.37	2.45 ± 0.15	7.52 ± 0.83	>80	10.37 ± 1.08	15.20 ± 0.72 ###
**19**	>80	>80	70.25 ± 3.06	>80	>80	68.00 ± 4.00	>80
**20**	31.20 ± 2.29	5.86 ± 0.40	7.90 ± 0.14	4.52 ± 0.43	24.33 ± 2.13	7.70 ± 0.32	9.92 ± 0.52 ^###^
**21**	>80	>80	>80	>80	>80	70.5 ± 4.86	>80
**22**	>80	63.25 ± 3.88	36.90 ± 0.64	36.90 ± 0.56	>80	51.30 ± 1.10	>80
**23**	73.50 ± 3.40	19.95 ± 2.89	15.93 ± 1.46	15.45 ± 1.35	61.65 ± 5.86	31.97 ± 1.76	31.70 ± 1.94 ^###^
**24**	>80	>80	>80	>80	>80	>80	>80
**25**	>80	>80	>80	>80	>80	>80	>80
**26**	4.00 ± 0.80	1.72 ± 0.12	1.73 ± 0.44	0.77 ± 0.03	4.46 ± 0.57	2.98 ± 0.10	4.22 ± 0.50 ^##^
**27**	1.22 ± 0.25	0.69 ± 0.08 ^*^	1.05 ± 0.07	0.52 ± 0.02	3.51 ± 0.15	0.50 ± 0.03	1.52 ± 0.12 ^#^
**28**	0.91 ± 0.03	0.66 ± 0.02 ^*^	0.83 ± 0.04	0.50 ± 0.06	1.70 ± 0.09	0.62 ± 0.03	1.61 ± 0.15 ^#^
**29**	0.63 ± 0.01 ^**^	0.85 ± 0.15	1.63 ± 0.15	0.79 ± 0.06	1.45 ± 0.07	6.64 ± 0.11	4.28 ± 0.33 ^###^
**30**	1.24 ± 0.09	1.72 ± 0.03	1.33 ± 0.07	0.44 ± 0.02	3.43 ± 0.28	1.03 ± 0.07	2.84 ± 0.34 ^#^
**31**	61.60 ± 1.73	49.17 ± 4.31	18.85 ± 1.34	13.73 ± 0.56	57.93 ± 2.60	30.23 ± 2.04	ND
**32**	0.74 ± 0.04 ^*^	0.81 ± 0.06	0.45 ± 0.01 ^*^	0.40 ± 0.02	1.08 ± 0.10	1.09 ± 0.09	1.70 ± 0.48
**33**	1.30 ± 0.08	1.00 ± 0.06	1.20 ± 0.09	0.53 ± 0.05	4.23 ± 0.39	0.57 ± 0.03	1.75 ± 0.09 ^#^
**34**	21.40 ± 3.71	8.65 ± 0.46	7.74 ± 1.43	>20	>80	5.63 ± 0.20	7.30 ± 1.20 ^#^
**35**	0.50 ± 0.05 ^**^	0.27 ± 0.05 ^**^	0.40 ± 0.02 ^**^	0.26 ± 0.01 ^**^	0.49 ± 0.02 ^***^	0.20 ± 0.01 ^***^	1.17 ± 0.41
**36**	1.29 ± 0.14	1.85 ± 0.08	0.56 ± 0.01	0.28 ± 0.03*	4.96 ± 0.88	2.45 ± 0.09	1.62 ± 0.08 ^#^
**GA**	0.94 ± 0.03	0.84 ± 0.04	0.59 ± 0.03	0.49 ± 0.03	1.14 ± 0.06	0.27 ± 0.01	0.89 ± 0.08

ND, not determined. ^a^ Data are the mean of three experiments. ^*^p < 0.05; ^**^p < 0.01; ^***^p < 0.005 compared with GA. ^#^* p* < 0.05; ^##^* p* < 0.01; ^###^ *p <* 0.005 compared with GA, representing survival rates.

Among them, the IC_50_ value for the parent compound (GA) was 0.94 μM in A549 cell line, 0.84 μM in HepG2 cell line, 0.59 μM in HCT116 cell line, 0.49 μM in K562 cell line, 1.14 μM in HeLa cell line and 0.27 μM in HUVEC cell line, respectively. Compound **36** showed similar cytotoxicity against the same cell lines, 1.29 μM in A549 cell line, 1.85 μM in HepG2 cell line, 0.56 μM in HCT116 cell line, 0.28 μM in K562 cell line, 4.96 μM in HeLa cell line and 2.45 μM in HUVEC cell line. Importantly, compound **36** showed relatively lower toxicities to LO2 cell line than GA.

### 2.6. Structure-Activity Relationship

As a result of zebrafish embryos assay, wound-healing assay, tube formation assay and MTT assay, we found that compared to the unsubstituted GA, introduction of electron-withdrawing groups (EWG; e.g., nitro for **1**, **5**, **8**, **14**; halogenations including chlorine, bromine and fluorine atoms for **2**, **17**, **19**, **24**; cyano for **12**; carbonyl for **5**, **11**, **15**, **16**, **20**, **23**) and electron-donating groups on the phenyl ring (EDG; e.g., methoxyl for **3**, **13**, **22**, **31**; 2-methoxyethyl for **18**; ethyl for **21**; 2,4,4-trimethylpentan-2-yl for **25**) failed to improve their antiangiogenic potencies in contrast to GA. The acetamido group at the *para* position of the phenyl ring (*para*-acetamido for **4** and **35**) improved the antiangiogenic activity. A heterocyclic ring substituent (thiazole for **32**) showed more potency compared with GA. These properties may contribute to the basic groups which can improve the solubility of the compounds in acidic media [22]. It has been reported that epoxidation of the carbon-carbon double bond at C-32/33, C-37/38 could improve antiangiogenic activity [21]. Therefore, we modified the carbon-carbon double bond on the structure of **35** to produce derivative **36**. We found that it indeed improved antiangiogenic activity.

## 3. Experimental

### 3.1. Chemistry

All commercial chemicals and solvents were reagent grade and were used without further treatment unless otherwise noted. Chemistry reagents of analytical grade were purchased from Chengdu Changzheng Chemical Factory, Sichuan, China. Analytical thin-layer chromatography was performed on 0.20 mm Silica Gel 60 F_254_ plates (Qingdao Ocean Chemical Factory, Shandong, China). Nuclear magnetic resonance spectra (NMR) were recorded at 400 MHz on a Varian model Gemini 400 spectrometer (Varian, Palo Alto, CA, USA) and peaks are reported in parts per million. Mass spectra (MS) were measured by a Q-TOF Premier mass spectrometer (Micromass, Manchester, UK). 

*General procedure for the synthesis of gambogates, gambogthioesters and gambogamides* **1–35**. A mixture of gambogic acid (0.18 mmol), R-OH or R-SH or R-NH_2_ (0.20 mmol), DMAP (0.10 mmol) and EDCI (0.30 mmol) in CH_2_Cl_2_ (10 mL) was stirred at 25 °C overnight. The solution was washed by hydrochloric acid solution (2 × 10 mL) and water (2 × 10 mL). The combined organic layer was dried and concentrated to yield the crude product, which was purified by silica gel column chromatography.

*4-Methyl-3-nitrophenyl gambogate* (**1**) Yield 70.6%; HPLC: 98.6%; yellow oil; ^1^H-NMR (CDCl_3_): δ 12.81 (s, 1H), 7.54 (q, *J* = 2.8 Hz, 1H), 7.32 (m, 1H), 6.64 (m, 2H), 6.41 (m, 1H), 5.45 (m, 1H), 5.05 (m, 2H), 3.48 (t, *J* = 5.6 Hz, 1H), 3.32 (m, 1H), 3.16 (d, *J* = 9.2 Hz, 1H), 3.03–2.89 (m, 2H), 2.56 (t, *J* = 7.2 Hz, 1H), 2.35 (m, 1H), 2.04 (m, 2H), 1.73 (m, 6H), 1.66 (s, 3H), 1.62 (d, *J* = 4.4 Hz, 3H), 1.58 (s, 3H), 1.55 (s, 3H), 1.47-1.38 (m, 3H), 1.35 (s, 1H), 1.30 (d, *J* = 6.4 Hz, 3H), 1.26 (s, 2H); MS (ESI, *m/z*): 819.29 [M − H]^−^. 

*6-Bromo-2,3-difluorophenyl gambogate* (**2**) Yield 67%; HPLC: 98.2%; yellow oil; ^1^H-NMR (CDCl_3_): δ 12.81 (s, 1H), 7.54 (q, *J* = 2.8 Hz, 1H), 7.32 (m, 1H), 6.64 (m, 2H), 6.41 (m, 1H), 5.45 (m, 1H), 5.05 (m, 2H), 3.48 (t, *J* = 5.6 Hz, 1H), 3.32 (m, 1H), 3.16 (d, *J* = 9.2 Hz, 1H), 3.03–2.89 (m, 2H), 2.56 (t, *J* = 7.2 Hz, 1H), 2.35 (m, 1H), 2.04 (m, 2H), 1.73 (m, 6H), 1.66 (s, 3H), 1.62 (d, *J* = 4.4 Hz, 3H), 1.58 (s, 3H), 1.55 (s, 3H), 1.47–1.38 (m, 3H), 1.35 (s, 1H), 1.30 (d, *J* = 6.4 Hz, 3H), 1.26 (s, 2H); MS (ESI, *m/z*): 819.29 [M − H]^−^. 

*4-Allyl-2-methoxyphenyl gambogate* (**3**)*.* Yield 75%; HPLC: 98.5%; brown oil; ^1^H-NMR (CDCl_3_): δ 12.82 (s, 1H), 7.48 (d, *J* = 7.2 Hz, 1H), 6.70 (m, 4H), 6.35 (s, 1H), 5.94 (m, 1H), 5.44 (m, 1H), 5.08 (m, 4H), 3.67 (d, *J* = 3.6 Hz, 3H), 3.44 (s, 1H), 3.35 (m, 3H), 3.19 (d, *J* = 14.4 Hz, 1H), 2.95 (m, 2H), 2.53 (t, *J* = 8.4 Hz, 1H), 2.3 (dd, *J* = 13.6 Hz, *J* = 4.8 Hz, 1H), 2.06 (m, 2H), 1.74 (m, 6H), 1.65 (m, 6H), 1.55 (s, 6H), 1.40 (m, 3H), 1.31 (m, 6H); MS (ESI, *m/z*):774.51 [M − H]^−^.

*4-Acetamidephenyl gambogate* (**4**). Yield 72%; HPLC: 99.1%; yellow oil; ^1^H-NMR (CDCl_3_): δ 12.83 (s, 1H), 7.52 (d, *J* = 7.2 Hz, 1H), 7.45 (d, *J* = 7.6 Hz, 2H), 7.12 (br, 1H), 6.83 (d, *J* = 7.6 Hz, 2H), 6.64 (m, 1H), 6.33 (m, 1H), 5.42 (t, *J* = 11.2 Hz, 1H), 5.05 (m, 2H), 3.47 (s, 1H), 3.34 (m, 1H), 3.16 (d, *J* = 11.2 Hz, 1H), 3.06 (m, 1H), 2.93 (m, 1H), 2.54 (d, *J* = 9.6 Hz, 1H), 2.32 (dd, *J* = 12.8 Hz, *J* = 3.2 Hz, 1H), 2.17 (s, 3H), 2.05 (m, 2H), 1.87 (d, *J* = 4 Hz, 3H), 1.73 (m, 6H), 1.63 (m, 6H), 1.55 (s, 3H), 1.41 (m, 3H), 1.32 (m, 3H); MS (ESI, *m/z*):762.42 [M + H]^+^.

*2-Nitro-4-ethanonephenyl gambogate* (**5**) Yield 63%; HPLC: 98.8%; Yellow oil; ^1^H NMR (CDCl_3_): δ 12.83 (s, 1H), 8.59 (m, 1H), 8.18 (m, 1H), 7.55 (t, *J* = 6.8 Hz, 1H), 7.46 (dd, *J* = 8.4 Hz, *J* = 2 Hz, 1H), 6.65 (m, 1H), 6.48 (m, 1H), 5.45 (m, 1H), 5.06 (m, 2H), 3.52 (m, 1H), 3.33 (m, 1H), 3.18 (m, 1H), 2.93 (m, 2H), 2.67 (t, 3H), 2.55 (m, 1H), 2.35 (m, 1H), 2.08 (m, 2H), 1.74 (m, 6H), 1.66 (m, 9H), 1.51 (s, 3H), 1.39 (m, 3H), 1.26 (m, 6H); MS (ESI, *m/z*:792.33) [M + H]^+^.

*Coumarin-3-gambogate* (**6**) Yield 52%; HPLC: 99.1%; buff-colored oil; ^1^H-NMR (CDCl_3_): δ 12.90 (s, 1H), 7.55 (d, *J* = 6.8 Hz), 7.50 (m, 2H), 7.31 (m, 3H), 6.65 (d, *J* = 10.4 Hz, 1H), 6.44 (m, 1H), 5.43 (m, 1H), 5.04 (m, 2H), 3.48 (m, 1H), 3.37 (m, 1H), 3.12 (m, 2H), 2.87 (m, 1H), 2.55 (d, *J* = 9.2 Hz, 1H), 2.32 (dd, *J* = 13.2 Hz, *J* = 4.4 Hz, 1H), 2.05 (m, 2H), 1.78 (m, 6H), 1.63 (m, 6H), 1.57 (m, 3H), 1.39 (m, 6H), 1.25 (m, 6H); MS (ESI, *m/z*): 773.79 [M + H]^+^.

*Coumarin-7-gambogate* (**7**) Yield 66%; HPLC: 99.2%; buff-colored oil; ^1^H-NMR (CDCl_3_): δ 12.81 (s, 1H), 7.66 (m, 1H), 7.54 (dd, *J* = 6.8 Hz, *J* = 2 Hz, 1H), 7.44 (m, 1H), 6.97 (d, *J* = 2 Hz, 1H), 6.83 (m, 1H), 6.32 (d, *J* = 10 Hz, 1H), 6.37 (m, 2H), 5.43 (m, 1H), 5.04 (br, 2H), 3.49 (s, 1H), 3.34 (m, 1H), 3.18 (m, 1H), 3.06 (m, 1H), 2.95 (m, 1H), 2.55 (d, *J* = 8.8 Hz, 1H), 2.33 (dd, *J* = 13.6 Hz, *J* = 4.4 Hz, 1H), 2.02 (m, 2H), 1.75 (m, 6H), 1.65 (m, 6H), 1.55 (m, 3H), 1.36 (m, 6H), 1.26 (m, 6H); MS (ESI, *m/z*): 773.79 [M + H]^+^.

*3-Nitrophenyl gambogate* (**8**) Yield 87%; HPLC: 99.1%; yellow oil; ^1^H-NMR (CDCl_3_): δ 12.79 (s, 1H), 8.06 (d, *J* = 7.6 Hz, 1H), 7.84 (s, 1H), 7.52 (m, 2H), 6.62 (d, *J* = 10 Hz, 1H), 6.36 (t, *J* = 6.8 Hz, 1H), 5.39 (d, *J* = 10 Hz, 1H), 5.03 (br, 2H), 3.49 (t, *J* = 5.2 Hz, 1H), 3.33 (dd, *J* = 14.4 Hz, *J* = 8 Hz, 1H), 3.17 (m, 1H), 3.06 (dd, *J* = 16.8 Hz, *J* = 8 Hz, 1H), 2.96 (dd, *J* = 16.8 Hz, *J* = 5.2 Hz, 1H), 2.56 (d, *J* = 8.8 Hz, 1H), 2.33 (dd, *J* = 13.2 Hz, *J* = 4.4 Hz, 1H), 2.01 (m, 2H), 1.73 (s, 3H), 1.72 (s, 3H), 1.64 (s, 3H), 1.62 (s, 3H), 1.56 (s, 3H), 1.37 (m, 6H), 1.30 (s, 3H), 1.25 (s, 3H); MS (ESI, *m/z*): 750.55 [M + H]^+^.

*3-Chloro-N-(4-phenylgambogate)propanamide* (**9**) Yield 39%; HPLC: 99.4%; yellow oil; ^1^H-NMR (CDCl_3_): δ 12.83 (s, 1H), 7.52 (d, *J* = 7.2 Hz, 1H), 7.44 (dd, *J* = 8.8 Hz, *J* = 2 Hz, 2H), 7.37 (d, *J* = 11.2 Hz, 1H), 6.83 (d, *J* = 8.4 Hz, 2H), 6.63 (m, 1H), 6.35 (m, 1H), 5.43 (m, 1H), 5.05 (m, 2H), 3.87 (t, *J* = 6.4 Hz, 2H), 3.47 (m, 1H), 3.34 (m, 1H), 3.17–3.04 (m, 2H), 2.90 (m, 1H), 2.78 (t, *J* = 6 Hz, 2H), 2.54 (d, *J* = 9.2 Hz, 1H), 2.31 (dd, *J* = 13.2 Hz, *J* = 4.4 Hz, 1H), 2.03 (m, 2H), 1.87 (s, 3H), 1.75 (m, 6H), 1.64 (m, 6H), 1.55 (s, 3H), 1.38 (s, 3H), 1.34 (s, 3H), 1.30 (s, 3H); MS (ESI, *m/z*): 810.53 [M + H]^+^.

*Geraniol gambogate* (**10**) Yield 39%; HPLC: 98.5%; yellow oil; ^1^H-NMR (CDCl_3_): δ 12.79 (s, 1H), 7.52 (t, *J* = 6.8 Hz, 1H), 6.68 (m, 1H), 6.32 (t, *J* = 4Hz, 1H), 5.45 (d, *J* = 9.2 Hz, 1H), 5.32 (t, *J* = 6.8Hz, 1H), 5.15 (m, 1H), 5.06 (m, 2H), 4.56 (m, 2H), 3.50 (m, 1H), 3.31 (m, 2H), 3.15–2.95 (m, 1H), 2.64 (d, *J* = 7.6 Hz, 2H), 2.52 (dd, *J* = 9.6 Hz, *J* = 4.0 Hz, 1H), 2.33 (m, 1H), 2.12–2.01 (m, 6H), 1.76 (m, 6H), 1.64 (m, 12H), 1.61(m, 6H), 1.58 (s, 3H), 1.43 (s, 3H), 1.38 (s, 3H), 1.29 (s, 3H); MS (ESI, *m/z*): 787.79 [M + Na]^+^.

*3-Ethanonephenyl gambogate* (**11**) Yield 91%; HPLC: 99.1%; yellow oil; ^1^H-NMR (CDCl_3_): δ 12.87 (s, 1H), 7.78 (d, *J* = 5.2 Hz, 1H), 7.52 (m, 2H), 7.42 (m, 1H), 7.08 (m, 1H), 6.63 (m, 1H), 6.40 (m, 1H), 5.41 (m, 1H), 5.03 (br, 2H), 3.47 (s, 1H), 3.35 (m, 1H), 3.17 (m, 1H), 3.07 (dd, *J* = 17.2 Hz, *J* = 8.4 Hz, 1H), 2.92 (m, 1H), 2.60 (s, 3H), 2.56 (m, 1H), 2.31 (dd, *J* = 13.6 Hz, *J* = 4.4 Hz, 1H), 2.02 (m, 2H), 1.90 (d, *J* = 4.4 Hz, 3H), 1.78 (m, 7H), 1.64 (m, 7H), 1.55 (m, 3H), 1.39 (m, 4H), 1.30 (s, 3H); MS (ESI, *m/z*): 745.62 [M − H]^−^.

*3-Cyanophenyl gambogate* (**12**) Yield 86%; HPLC: 99.3%; brown oil; ^1^H-NMR (CDCl_3_): δ 12.83 (s, 1H), 7.54 (d, *J* = 5.2 Hz, 1H), 7.44 (m, 2H), 7.23 (m, 1H), 7.14 (m, 1H), 6.40 (dd, *J* = 10.4 Hz, *J* = 6.0 Hz, 1H), 6.43–6.32 (m, 1H), 5.44 (dd, *J* = 12.4 Hz, *J* = 10.4 Hz, 1H), 5.04 (m, 2H), 3.48 (m, 1H), 3.34 (m, 1H), 3.13 (m, 1H), 3.04 (m, 1H), 2.97–2.86 (m, 1H), 2.55 (d, *J* = 9.2 Hz, 1H), 2.32 (m, 1H), 2.01 (m, 2H), 1.89 (d, *J* = 8.0 Hz, 3H), 1.74 (d, *J* = 4.0 Hz, 4H), 1.72 (d, *J* = 3.2 Hz, 3H), 1.64 (m, 6H), 1.54 (d, *J* = 4.4 Hz, 3H), 1.36 (m, 3H), 1.30 (s, 3H), 1.28 (s, 2H); MS (ESI, *m/z*): 752.45 [M + Na]^+^.

*4-Methoxyphenyl gambogate* (**13**) Yield 92%; HPLC: 99.5%; brown oil; ^1^H-NMR (CDCl_3_): δ 12.86 (s, 1H), 7.53 (d, *J* = 6.4 Hz, 1H), 6.80 (m, 4H), 6.66 (d, *J* = 10.0 Hz, 1H), 6.32 (t, *J* = 6.4 Hz, 1H), 5.42 (d, *J* = 10.4 Hz, 1H), 5.05 (m, 2H), 3.79 (s, 3H), 3.48 (m, 1H), 3.34 (m, 1H), 3.18 (m, 1H), 3.08 (m, 1H), 2.94 (m, 1H), 2.55 (d, *J* = 9.2 Hz, 1H), 2.32 (m, 1H), 2.05 (m, 2H), 1.88 (s, 3H), 1.78 (m, 6H), 1.63 (m, 6H), 1.58 (s, 3H), 1.40 (s, 3H), 1.31 (m, 6H); MS (ESI, *m/z*): 733.77 [M − H]^−^.

*3-Methyl-2-nitrophenyl gambogate*(**14**) Yield 78%; HPLC: 99.1%; yellow powder; ^1^H-NMR (CDCl_3_): δ 12.86 (s, 1H), 7.53 (d, *J* = 7.2 Hz, 1H), 7.34 (t, *J* = 8.0 Hz, 1H), 7.10 (d, *J* = 8.0 Hz, 1H), 6.90 (8.4 Hz, 1H), 6.60 (d, *J* = 10.0 Hz, 1H), 6.41 (t, *J* = 6.0 Hz, 1H), 5.38 (d, *J* = 10.0 Hz, 1H), 5.04 (m, 2H), 3.47 (m, 1H), 3.33 (dd, *J* = 10.4 Hz, *J* = 8.0 Hz, 1H), 3.16 (dd, *J* = 14.4 Hz, *J* = 4.8 Hz, 1H), 2.99 (m, 1H), 2.88 (m, 1H), 2.53 (d, *J* = 9.2 Hz, 1H), 2.32 (s, 3H), 2.29 (d, *J* = 4.8 Hz, 1H), 2.01 (m, 2H), 1.83 (s, 3H), 1.74 (s, 3H), 1.71 (s, 3H), 1.65 (s, 3H), 1.62 (s, 3H), 1.55 (s, 3H), 1.38 (s, 3H), 1.29 (s, 3H), 1.25 (s, 3H); MS (ESI, *m/z*): 733.40 [M + H]^+^.

*3-Formylphenyl gambogate* (**15**) Yield 87%; HPLC: 98.3%; brown oil; ^1^H-NMR (CDCl_3_): δ 12.84 (s, 1H), 9.98 (s, 1H), 7.72 (d, *J* = 7.6 Hz, 1H), 7.53 (d, *J* = 6.8 Hz, 1H), 7.48 (t, *J* = 8.0 Hz, 1H), 7.42 (s, 1H), 7.15 (d, *J* = 8.0 Hz, 1H), 6.62 (d, *J* = 10.0 Hz, 1H), 6.37 (t, *J* = 6.8 Hz, 1H), 5.39 (d, *J* = 10.0 Hz, 1H), 5.04 (s, 2H), 3.50 (t, *J* = 4.8 Hz, 1H), 3.33 (m, 1H), 3.14 (m, 1H), 3.08 (t, *J* = 8.0 Hz, 1H), 2.94 (dd, *J* = 16.8 Hz, *J* = 4.4 Hz, 1H), 2.55 (d, *J* = 9.6 Hz, 1H), 2.32 (dd, *J* = 13.6 Hz, *J* = 4.8 Hz, 1H), 2.01 (m, 2H), 1.90 (s, 3H), 1.73 (s, 3H), 1.72 (s, 3H), 1.65 (s, 3H), 1.62 (s, 3H), 1.55 (s, 3H), 1.35 (s, 3H), 1.31 (s, 3H), 1.26 (s, 3H); MS (ESI, *m/z*): 733.40 [M + H]^+^.

*4-(2-Bromoacetyl)phenyl gambogate* (**16**) Yield 73%; HPLC: 98.9%; yellow oil; ^1^H-NMR (CDCl_3_): δ 12.83 (s, 1H), 7.93 (d, *J* = 8.4 Hz, 2H), 7.53 (d, *J* = 7.2 Hz, 1 H), 7.02 (d, *J* = 8.4 Hz, 2H), 6.62 (d, *J* = 10.0 Hz, 1H), 6.36 (t, *J* = 6.4 Hz, 1H), 5.40 (d, *J* = 10.0 Hz, 1H), 5.04 (m, 2H), 4.69 (s, 2H), 3.48 (m, 1H), 3. 31 (dd, *J* = 14.8 Hz, *J* = 7.6 Hz, 1H), 3.16 (m, 1H), 3.06 (m, 1H), 2.97 (m, 1H), 2.55 (d, *J* = 9.6 Hz, 1H), 2.33 (dd, *J* = 13.2 Hz, *J* = 4.4 Hz, 1H), 2.01 (m, 2H), 1.88 (s, 3H), 1.73 (s, 3H), 1.72 (s, 3H), 1.65 (s, 3H), 1.62 (s, 3H), 1.56 (s, 3H), 1.36 (m, 4H), 1.27 (m, 5H); MS (ESI, *m/z*): 825.78 [M + H]^+^.

*2-Bromophenyl gambogate* (**17**) Yield 88%; HPLC: 98.5%; brown oil; ^1^H-NMR (CDCl_3_): δ 12.86 (s, 1H), 7.52 (m, 2H), 7.27 (m, 1H), 7.05 (m, 1H), 6.94 (dd, *J* = 8.0 Hz, *J* = 1.2 Hz, 1H), 6.64 (d, *J* = 10.0 Hz, 1H), 6.46 (t, *J* = 6.4 Hz, 1H), 5.41 (d, *J* = 10.0 Hz, 1H), 5.05 (br, 2H), 3.46 (m, 1H), 3.35 (dd, *J* = 14.4 Hz, *J* = 8.0 Hz, 1H), 3.20 (m, 1H), 3.06 (dd, *J* = 17.6 Hz, *J* = 7.2 Hz, 1H), 2.96 (m, 1H), 2.54 (d, *J* = 9.2 Hz, 1H), 2.31 (dd, *J* = 13.6 Hz, *J* = 4.4 Hz, 1H), 2.04 (m, 2H), 1.95 (s, 3H), 1.75 (s, 3H), 1.73 (s, 3H), 1.65 (s, 3H), 1.63 (s, 3H), 1.55 (s, 3H), 1.40 (m, 5H), 1.28 (m, 4H); MS (ESI, *m/z*): 783.42 [M + H]^+^.

*4-(2-Methoxyethyl)phenyl* gambogate (**18**). Yield 82%; HPLC: 99.0%; brown powder; ^1^H-NMR (CDCl_3_): δ 12.84 (s, 1H), 7.51 (d, *J* = 7.2 Hz, 1H), 7.15 (d, *J* = 8.0 Hz, 2H), 6.78 (d, *J* = 8.4 Hz, 2H), 6.65 (d, *J* = 10.0 Hz, 1H), 6.31 (t, *J* = 6.4 Hz, 1H), 5.41 (d, *J* = 10.0 Hz, 1H), 5.05 (br, 2H), 3.56 (t, *J* = 7.2 Hz, 2H), 3.46 (m, 1H), 3.35 (s, 3H), 3.31 (m, 1H), 3.18 (m, 1H), 3.03 (m, 1H), 2.94 (m, 1H), 2.84 (t, 7.2 Hz, 2H), 2.53 (d, *J* = 9.2 Hz, 1H), 2.31 (dd, *J* = 13.6 Hz, *J* = 4.8 Hz, 1H), 2.03 (m, 2H), 1.86 (s, 3H), 1.73 (s, 3H), 1.71 (s, 3H), 1.65 (s, 3H), 1.62 (s, 3H), 1.55 (s, 3H), 1.38 (m, 5H), 1.29 (m, 4H); MS (ESI, *m/z*): 785.78 [M + Na]^+^.

*4-Bromophenyl gambogate* (**19**) Yield 92%; HPLC: 99.2%; yellow oil; ^1^H-NMR (CDCl_3_): δ 12.83 (s, 1H), 7.52 (d, *J* = 6.8 Hz, 1H), 7,41 (d, *J* = 8.4 Hz, 2H), 6.76 (d, *J* = 8.8 Hz, 2H), 6.63 (d, *J* = 10.4 Hz, 1H), 6.29 (t, *J* = 6.8 Hz, 1H), 5.41 (d, *J* = 10.0 Hz, 1H), 5.04 (s, 2H), 3.47 (t, *J* = 5.2 Hz, 1H), 3.31 (dd, *J* = 14.0 Hz, 1H), 3.17 (m, 1H), 3.05 (dd, *J* = 17.2 Hz, *J* = 7.6 Hz, 1H), 2.97 (m, 1H), 2.54 (d, *J* = 9.6 Hz, 1H), 2.31 (dd, *J* = 13.6 Hz, *J* = 4.4 Hz, 1H), 2.03 (m, 2H), 1.86 (s, 3H), 1.76 (s, 3H), 1.73 (s, 3H), 1.65 (s, 3H), 1.62 (s, 3H), 1.57 (s, 3H), 1.37 (m, 5H), 1.25 (m, 4H); MS (ESI, *m/z*): 805.62 [M + Na]^+^.

*4-Formylphenyl gambogate* (**20**) Yield 89%; HPLC: 99.5%; brown oil; ^1^H-NMR (CDCl_3_): δ 12.82 (s, 1H), 9.96 (s, 1H), 7.85 (d, *J* = 8.4 Hz, 2H), 7.52 (d, *J* = 6.8 Hz, 1H), 7.05 (d, *J* = 8.4 Hz, 2H), 6.61 (d, *J* = 10.0 Hz, 1H), 6.33 (t, *J* = 6.8 Hz, 1H), 5.39 (d, *J* = 10.4 Hz, 1H), 5.03 (br, 2H), 3.48 (t, *J* = 5.6 Hz, 1H), 3.31 (dd, *J* = 14.8 Hz, *J* = 8.0 Hz, 1H), 3.17 (m, 1H), 3.06 (m, 1H), 2.96 (dd, *J* = 17.2 Hz, *J* = 5.2 Hz, 1H), 2.54 (d, *J* = 9.2 Hz, 1H), 2.32 (dd, *J* = 13.2 Hz, *J* = 4.4 Hz, 1H), 2.01 (m, 2H), 1.88 (s, 3H), 1.72 (s, 3H), 1.71 (s, 3H), 1.64 (s, 3H), 1.61 (s, 3H), 1.57 (s, 3H), 1.39 (m, 5H), 1.29 (m, 4H); MS (ESI, *m/z*): 755.61 [M + Na]^+^.

*4-Ethylphenyl gambogate* (**21**) Yield 87%; HPLC: 99.3%; yellow oil; ^1^H-NMR (CDCl_3_): δ 12.83 (s, 1H), 7.51 (d, *J* = 6.8 Hz, 1H), 7.12 (d, *J* = 8.0 Hz, 2H), 6.77 (d, *J* = 8.0 Hz, 2H), 6.66 (d, *J* = 10.0 Hz, 1H), 6.31 (t, *J* = 6.8 Hz, 1H), 5.41 (d, *J* = 10.4 Hz, 1H), 5.04 (br, 2H), 3.46 (t, *J* = 5.6 Hz, 1H), 3.32 (dd, *J* = 14.4 Hz, *J* = 8.0 Hz, 1H), 3.17 (m, 1H), 3.06 (dd, *J* = 16.8 Hz, *J* = 7.6 Hz, 1H), 2.93 (dd, 16.4 Hz, *J* = 5.6 Hz, 1H), 2.61 (m, 2H), 2.54 (d, *J* = 8.8 Hz, 1H), 2.31 (dd, *J* = 13.2 Hz, *J* = 4.4 Hz, 1H), 2.02 (m, 2H), 1.87 (s, 3H), 1.73 (s, 3H), 1.72 (s, 3H), 1.65 (s, 3H), 1.62 (s, 3H), 1.55 (s, 3H), 1.41 (m, 5H), 1.30 (m, 4H), 1.20 (t, *J* = 7.6 Hz, 3H); MS (ESI, *m/z*): 755.56 [M + Na]^+^.

*2-Bromo-4-methoxyphenyl gambogate* (**22**) Yield 73%; HPLC: 98.7%; brown oil; ^1^H-NMR (CDCl_3_): δ 12.85 (s, 1H), 7.52 (d, *J* = 6.8 Hz, 1H), 7.04 (d, *J* = 2.8 Hz, 1H), 6.80 (m, 2H), 6.63 (d, *J* = 10.0 Hz, 1H), 6.44 (t, *J* = 6.8 Hz, 1H), 5.41 (d, *J* = 10.0 Hz, 1H), 5.05 (br, 2H), 3.75 (s, 3H), 3.46 (m, 1H), 3.33 (m, 1H), 3.20 (m, 1H), 3.03 (m, 1H), 2.95 (m, 1H), 2.53 (d, *J* = 9.2 Hz, 1H), 2.30 (dd, *J* = 13.6 Hz, *J* = 4.8 Hz, 1H), 2.01 (m, 2H), 1.93 (s, 3H), 1.74 (s, 3H), 1.72 (s, 3H), 1.65 (s, 3H), 1.63 (s, 3H), 1.55 (s, 3H), 1.40 (m, 5H), 1.29 (m, 4H); MS (ESI, *m/z*): 835.56 [M + Na]^+^.

*4-Chloro-2-formylphenyl gambogate* (**23**) Yield 69%; HPLC: 99.6%; brown oil; ^1^H-NMR (CDCl_3_): δ 12.81 (s, 1H), 9.95 (s, 1H), 7.78 (d, *J* = 2.4 Hz, 1H), 7.52 (m, 2H), 6.98 (d, *J* = 8.4 Hz, 1H), 6.57 (d, *J* = 10.0 Hz, 1H), 6.38 (t, *J* = 6.4 Hz, 1H), 5.39 (d, *J* = 10.0 Hz, 1H), 5.03 (br, 2H), 3.48 (m, 1H), 3.31 (dd, *J* = 14.4 Hz, *J* = 8.0 Hz, 1H), 3.17 (m, 1H), 3.01 (m, 2H), 2.54 (d, *J* = 8.8 Hz, 1H), 2.32 (dd, *J* = 13.2 Hz, *J* = 4.4 Hz, 1H), 2.02 (m, 2H), 1.98 (s, 3H), 1.78 (s, 3H), 1.71 (s, 3H), 1.64 (s, 3H), 1.62 (s, 3H), 1.54 (s, 3H), 1.38 (m, 4H), 1.30 (m, 5H); MS (ESI, *m/z*): 767.25 [M + Na]^+^.

*4-Bromo-2,3-difluorophenyl gambogate* (**24**) Yield 72%; HPLC: 99.3%; brown oil; ^1^H-NMR (CDCl_3_): δ 12.80 (s, 1H), 7.53 (d, *J* = 7.2 Hz, 1H), 7.27 (m, 1H), 6.69 (m, 1H), 6.62 (d, *J* = 10.4 Hz, 1H), 6.40 (t, *J* = 6.8 Hz, 1H), 5.42 (d, *J* = 10.0 Hz, 1H), 5.04 (m, 2H), 3.48 (m, 1H), 3.31 (dd, *J* = 14.4 Hz, *J* = 8 Hz, 1H), 3.16 (dd, *J* = 14.4 Hz, *J* = 4.8 Hz, 1H), 2.95 (m, 2H), 2.54 (d, *J* = 9.2 Hz, 1H), 2.32 (dd, *J* = 13.2 Hz, *J* = 4.4 Hz, 1H), 2.02 (m, 2H), 1.90 (s, 3H), 1.76 (s, 3H), 1.71 (s, 3H), 1.65 (s, 3H), 1.62 (s, 3H), 1.57 (s, 3H), 1.38 (m, 5H), 1.27 (m, 4H); MS (ESI, *m/z*): 841.48 [M + Na]^+^.

*4-(2,4,4-Trimethylpentan-2-yl)phenyl gambogate* (**25**) Yield 81%; HPLC: 99.2%; yellow powder; ^1^H-NMR (CDCl_3_): δ 12.84 (s, 1H), 7.54 (d, *J* = 6.8 Hz, 1H), 7.28 (d, *J* = 8.8 Hz, 2H), 6.77 (d, *J* = 8.8 Hz, 2H), 6.66 (dd, *J* = 10.0 Hz, *J* = 3.6 Hz, 1H), 6.31 (t, *J* = 6.4 Hz, 1H), 5.42 (m, 1H), 5.05 (m, 2H), 3.52 (m, 1H), 3.36 (dd, *J* = 15.2 Hz, *J* = 8.0 Hz, 1H), 3.20 (m, 1H), 3.08 (m, 1H), 2.97 (dd, *J* = 17.2 Hz, *J* = 4.8 Hz, 1H), 2.55 (m, 1H), 2.33 (m, 1H), 2.04 (m, 2H), 1.88 (s, 3H), 1.75 (m, 12H), 1.65 (s, 3H), 1.62 (s, 3H), 1.58 (s, 3H), 1.41 (m, 6H), 1.36 (m, 6H), 1.27 (m, 8H); MS (ESI, *m/z*): 839.99 [M + Na]^+^.

*4-Butyramidophenyl gambogate* (**26**) Yield 63%; HPLC: 98.6%; yellow powder; ^1^H-NMR (CDCl_3_): δ 12.83 (s, 1H), 7.51 (d, *J* = 7.2 Hz, 1H), 7.44 (d, *J* = 8.8 Hz, 2H), 7.29 (s, 1H), 6.81 (d, *J* = 8.8 Hz, 2H), 6.64 (m, 1H), 6.32 (t, *J* = 6.8 Hz, 1H), 5.40 (d, 10.0 Hz, 1H), 5.04 (m, 2H), 3.46 (m, 1H), 3. 33 (dd, *J* = 14.4 Hz, *J* = 8.0 Hz, 1H), 3.15 (m, 1H), 3.07 (m, 1H), 2.91 (dd, *J* = 17.2 Hz, *J* = 4.4 Hz, 1H), 2.54 (d, *J* = 9.2 Hz, 1H), 2.33 (m, 3H), 2.01 (m, 2H), 1.86 (s, 3H), 1.76 (m, 8H), 1.62 (m, 6H), 1.55 (s, 3H), 1.38 (m, 4H), 1.30 (m, 5H), 1.00 (t, *J* = 7.6 Hz, 3H); MS (ESI, *m/z*): 812.78 [M + Na]^+^.

*2-Acetamidophenyl gambogate* (**27**) Yield 67%; HPLC: 98.3%; yellow oil; ^1^H-NMR (CDCl_3_): δ 12.86 (s, 1H), 8.17 (d, *J* = 7.6 Hz, 1H), 7.49 (d, *J* = 6.8 Hz, 1H), 7.46 (s, 1H), 7.17 (t, *J* = 7.2 Hz, 1H), 7.05 (t, *J* = 7.2 Hz, 1H), 6.96 (dd, *J* = 8.4 Hz, *J* = 1.2 Hz, 1H), 6. 61 (d, *J* = 10.4 Hz, 1H), 6.09 (7.2 Hz, 1H), 5.39 (d, *J* = 10.0 Hz, 1H), 5.05 (m, 2H), 3.47 (m, 1H), 3.32–3.21 (m, 2H), 3.06–2.99 (m, 2H), 2.54 (d, *J* = 9.2 Hz, 1H), 2.32 (dd, *J* = 13.2 Hz, *J* = 4.4 Hz, 1H), 2.17 (s, 3H), 2.05 (m, 2H), 1.91 (s, 3H), 1.76 (s, 3H), 1.71 (s, 3H), 1.65 (s, 3H), 1.62 (s, 3H), 1.58 (s, 3H), 1.40 (m, 1H), 1.37 (s, 3H), 1.28 (m, 4H); MS (ESI, *m/z*): 784.71 [M + Na]^+^.

*3-Acetamidophenyl gambogate* (**28**) Yield 73%; HPLC: 99.4%; yellow oil; ^1^H-NMR (CDCl_3_): δ 13.19 (s, 1H), 7.71 (d, *J* = 8.0 Hz, 1H), 7.56 (m, 2H), 7.23 (d, *J* =8.4 Hz, 1H), 6.61 (m, 2H), 6.51 (s, 1H), 6.47 (dd, *J* = 7.6 Hz, *J* = 5.2 Hz, 1H), 5.42 (d, *J* = 10.0 Hz, 1H), 5.04 (m, 2H), 3.48 (m, 1H), 3.35 (dd, *J* = 14.8 Hz, *J* = 8.4 Hz, 1H), 3.15 (m, 2H), 2.78 (m, 1H), 2.54 (d, *J* = 9.2 Hz, 1H), 2.32 (dd, *J* = 13.6 Hz, *J* = 4.8 Hz, 1H), 2.19 (s, 3H), 2.01 (q, *J* = 8.0 Hz, 2H), 1.89 (s, 3H), 1.77 (s, 3H), 1.73 (s, 3H), 1.65 (s, 3H), 1.63 (s, 3H), 1.60 (s, 1H), 1.55 (s, 3H), 1.40 (m, 2H), 1.30 (s, 3H), 1.29 (s, 3H); MS (ESI, *m/z*): 784.79 [M + Na]^+^.

*S-thiazol-2-yl gambogthioate* (**29**) Yield 68%; HPLC: 99.2%; yellow oil; ^1^H-NMR (CDCl_3_): δ 12.79 (s, 1H), 7.89 (d, *J* = 3.6 Hz, 1H), 7.61 (d, *J* = 6.8 Hz, 1H), 7.51 (d, *J* = 3.2 Hz, 1H), 6.69 (m, 2H), 5.44 (d, *J* = 10.0 Hz, 1H), 5.07 (m, 2H), 3.54 (m, 1H), 3.25 (d, *J* = 6.8 Hz, 2H), 2.69 (dd, *J* = 15.6 Hz, *J* = 7.6 Hz, 1H), 2.61 (d, *J* = 7.2 Hz, 1H), 2.56 (d, *J* = 9.6 Hz, 1H), 2.36 (dd, *J* = 13.2 Hz, *J* = 4.8 Hz, 1H), 2.02 (m, 2H), 1.73 (s, 3H), 1.70 (s, 3H), 1.63 (m, 7H), 1.55 (s, 3H), 1.47 (s, 3H), 1.40 (m, 5H), 1.31 (s, 3H); MS (ESI, *m/z*): 728.54 [M + Na]^+^.

*4-Acetamidophenyl gambogthioate* (**30**) Yield 59%; HPLC: 99.5%; yellow oil; ^1^H-NMR (CDCl_3_): δ 12.89 (s, 1H), 7.59 (m, 1H), 7.48 (d, *J* = 6.8 Hz, 1H), 7.45 (d, *J* = 8.4 Hz, 2H), 7.15 (d, *J* = 8.4 Hz, 2H), 6.67 (d, *J* = 8.0 Hz, 1H), 6.02 (t, *J* = 6.4 Hz, 1H), 5.42 (m, 1H), 5.05 (m, 2H), 3.44 (m, 1H), 3.36 (m, 1H), 3.15 (m, 1H), 2.90 (dd, *J* = 17.2 Hz, *J* = 8.0 Hz, 1H), 2.79 (m, 1H), 2.55 (dd, *J* = 18.0 Hz, *J* = 9.2 Hz, 1H), 2.28 (dd, *J* = 13.2 Hz, *J* = 4.4 Hz, 1H), 2.09 (s, 3H), 2.04 (m, 2H), 1.93 (s, 3H), 1.75 (m, 3H), 1.69 (s, 3H), 1.65 (s, 3H), 1.62 (s, 3H), 1.55 (s, 3H), 1.47 (s, 3H), 1.39 (m, 3H), 1.27 (s, 3H); MS (ESI, *m/z*): 800.63 [M + Na]^+^.

*4-(4-Methoxyphenyl)thiazol-2-gambogamide* (**31**) Yield 59%; HPLC: 99.2%; yellow powder; ^1^H-NMR (CDCl_3_): δ 12.82 (s, 1H), 10.28 (s, 1H), 7.75 (d, *J* = 8.8 Hz, 2H), 7.61 (q, *J* = 3.6 Hz, 1H), 7.00 (d, *J* = 4.4 Hz), 6.91 (d, *J* = 8.8 Hz, 2H), 6.58 (dd, *J* = 10 Hz, *J* = 1.6 Hz, 1H), 5.77 (t, *J* = 8 Hz, 1H), 5.36 (q, *J* = 10 Hz, 1H), 5.10–4.89 (m, 2H), 3.84 (s, 3H), 3.53 (m, 1H), 3.07 (m, 2H), 2.76 (m, 2H), 2.60 (m, 1H), 2.37 (dd, *J* = 13.2 Hz, *J* = 4.4 Hz, 1H), 2.05 (m, 2H), 1.95 (m, 3H), 1.61 (m, 9H), 1.49 (m, 8H), 1.41 (m, 3H), 1.38 (s, 1H), 1.27 (m, 3H); MS (ESI, *m/z*): 817.52 [M + H]^+^.

*Thiazol-2-gambogamide* (**32**) Yield 58%; HPLC: 99.4%; yellow oil; ^1^H-NMR (CDCl_3_): δ 12.82 (s, 1H), 10.20 (br, 1H), 7.57 (dd, *J* = 7.2 Hz, *J* = 2.8 Hz, 1H), 7.42 (d, *J* = 3.6 Hz, 1H), 6.93 (m, 1H), 6.61 (q, *J* = 4.8 Hz, 1H), 5.65 (q, *J* = 8 Hz, 1H), 5.42 (q, *J* = 10 Hz, 1H), 5.12–4.98 (m, 2H), 3.52 (t, *J* = 4.8 Hz, 1H), 3.15 (m, 2H), 2.72 (m, 2H), 2.56 (m, 1H), 2.36 (m, 1H), 2.06 (m, 2H), 1.92 (s, 3H), 1.78 (m, 3H), 1.64 (s, 3H), 1.61 (m, 6H), 1.54 (s, 1H), 1.41 (s, 2H), 1.37 (m, 3H), 1.21 (m, 6H); MS (ESI, *m/z*): 711.54 [M + H]^+^.

*1-Gambogyl pyrazole* (**33**) Yield 49%; HPLC: 99.4%; yellow powder; ^1^H-NMR (CDCl_3_): δ 12.89 (s, 1H), 8.05 (m, 1H), 7.59 (m, 1H), 7.51 (m, 2H), 6.68 (dd, *J* = 20.8 Hz, *J* = 9.6 Hz, 1H), 6.33 (m, 1H), 5.44 (m, 1H), 5.06 (m, 2H), 3.46 (m, 1H), 3.43 (m, 1H), 3.24 (m, 3H), 2.48 (m, 1H), 2.30 (m, 1H), 2.03 (m, 2H), 1.90 (s, 3H), 1.75 (m, 3H), 1.65 (m, 9H), 1.55 (s, 3H), 1.43 (m, 3H), 1.25 (m, 6H); MS (ESI, *m/z*): 679.29 [M + H]^+^.

*N-(benzo[d]thiazol-2-yl) gambogamide* (**34**) Yield 59%; HPLC: 99.7%; yellow powder; ^1^H-NMR (CDCl_3_): δ 12.82 (s, 1H), 10.04 (br, 1H), 7.80 (d, *J* = 7.6 Hz, 1H), 7.72 (d, *J* = 8.0 Hz, 1H), 7.58 (d, *J* = 6.8 Hz, 1H), 7.40 (m, 1H), 7.30 (m, 1H), 6.52 (d, *J* = 10.4 Hz, 1H), 5.73 (m, 1H), 5.25 (dd, *J* = 10.4 Hz, *J* = 4.0 Hz, 1H), 4.96 (m, 2H), 3.53 (m, 1H), 3.10 (m, 2H), 2.81 (d, *J* = 8.8 Hz, 2H), 2.57 (d, *J* = 9.2 Hz, 1H), 2.37 (dd, *J* = 13.6 Hz, *J* = 4.4 Hz, 1H), 1.95 (m, 2H), 1.85 (s, 3H), 1.68 (m, 3H), 1.61 (s, 3H), 1.57 (s, 3H), 1.50 (s, 3H), 1.45 (s, 3H), 1.38 (m, 5H), 1.23 (m, 4H); MS (ESI, *m/z*): 761.69 [M + H]^+^.

*N-(4-acetamidophenyl) gambogamide* (**35**) Yield 73%; HPLC: 98.6%; yellow powder; ^1^H-NMR (CDCl_3_): δ 12.85 (s, 1H), 8.80 (s, 1H), 7.56 (m, 3H), 7.43 (d, *J* = 9.2 Hz, 2H), 7.38 (s, 1H), 6.67 (d, *J* = 10.4 Hz, 1H), 5.46 (d, *J* = 10.4 Hz, 1H), 5.31 (t, *J* = 8.8 Hz, 1H), 5.04 (m, 2H), 3.52 (m, 1H), 3.29 (dd, *J* = 14.8 Hz, *J* = 8.0 Hz, 1H), 3.14 (m, 1H), 2.81 (dd, *J* = 15.2 Hz, *J* = 9.2 Hz, 1H), 2.57 (d, *J* = 9.2 Hz, 1H), 2.34 (m, 2H), 2.15 (s, 3H), 2.02 (m, 2H), 1.89 (s, 3H), 1.80 (m, 2H), 1.70 (m, 6H), 1.65 (s, 3H), 1.62 (s, 3H), 1.55 (s, 3H), 1.44 (m, 4H), 1.36 (s, 3H); MS (ESI, *m/z*): 761.83 [M + H]^+^.

*(32,33),(37,38)-Diepoxy-N-(4-acetamidophenyl) gambogamide* (**36**). To a stirred solution of **35** (31 mg, 0.04 mmol) in CH_2_Cl_2_ (5 mL) at 25 °C, *m*-CPBA (16 mg, 0.09 mmol) was added. The resulting mixture was stirred for 6 h at 25 °C. The mixture was condensed. The residue was subjected to silica gel column chromatography (petrolum ether-acetone = 3:1) to provide **36**. Yield 71%; HPLC: 99.4%; yellow oil; ^1^H-NMR (CDCl_3_): δ 12.89 (s, 1H), 8.75 (s, 1H), 7.60–7.39 (m, 6H), 6.70 (m, 1H), 5.47 (m, 1H), 5.31 (m, 1H), 3.53 (t, *J* = 5.2 Hz, 1H), 2.90–2.70 (m, 6H), 2.60 (m, 2H), 2.34 (m, 2H), 2.16 (s, 3H), 1.87 (s, 3H), 1.65 (m, 6H), 1.47 (s, 3H), 1.35 (m, 6H), 1.28 (m, 9H); MS (ESI, *m/z*): 815.53 [M + Na]^+^.

### 3.2. Biological General Methods

#### 3.2.1. Zebrafish Embryo Assay

Using transgenic *fli-1*: enhanced GFP zebrafish embryos, we investigated the effects of all compounds on angiogenesis. Zebrafish embryos were generated by natural pairwise mating and raised at 28.5 °C in embryo water (0.2 g/L of Instant Ocean Salt in distilled water). At about 6 h post fertilization (hpf), the embryos were sorted in the 6-well plate (six embryos/well), removing dead and unhealthy embryos. Then the embryos were treated with the indicated concentrations of compounds which were added into embryo water. After incubation for 24 or 48 h, the embryos were anesthetized using 0.05% 2-phenoxyethanol in embryo water, quantified and photographed.

#### 3.2.2. Cell Culture

HepG2, HCT116, HeLa and LO2 cell lines were cultured in DMEM containing 10% fetal bovine serum (FBS), 100 IU/mL penicillin and 100 g/mL streptomycin; A549 and K562 cell lines were cultured in RPMI 1640 medium containing 10% fetal bovine serum (FBS), 100 IU/mL penicillin and 100 g/mL streptomycin; Human umbilical vein endothelial cells (HUVECs) were cultured on gelatin-coated culture flasks in M-199 medium with 20% fetal bovine serum (FBS), 2 ng/mL VEGF, 10 ng/mL basic FGF, 100 IU/mL penicillin, and 100 μg/mL streptomycin; all of them were obtained from the American Type Culture Collection (ATCC) and all cell lines were incubated in an atmosphere of 5% CO_2_ at 37 °C.

#### 3.2.3. Wound-Healing Assay

HUVECs were seeded in 6-well plates precoated with 0.1% gelatin, and grown overnight to confluence. The monolayer cells were wounded by scratching with 10 μL pipet tips and washed twice with serum-free DMEM (Dulbecco’s modified eagle medium) to remove the nonadherent cells and then replaced by serum-free DMEM with the indicated concentrations of compounds for 24 h. Images were taken at 0 h and 24 h independently after incubation at 37 °C, 5% CO_2_ environment. The migrated HUVECs were manually counted. The values were observed from four randomly selected fields.

#### 3.2.4. Tube Formation Assay

Matrigel was dissolved at 4 °C overnight. Each well of prechilled 96-well plates was coated with 50 μL of Matrigel, incubated and solidified at 37 °C for 45 min. After removing the unsolidified fluid, HUVECs at the density of 1 × 10^4^ were cultured in DMEM containing the indicated concentrations of compounds for 6 h. Controls were treated with the DMEM alone. Images were digitally captured and quantitatively analyzed (Olympus). The numbers and line lengths of the circular tubules formed by the cells were calculated manually. The values were observed from five randomly selected fields.

#### 3.2.5. MTT Assay

Cells were treated with various concentrations of compound in 96-well culture plates for 24 h in final volumes of 200 μL (5 × 10^3^ cells/well). Then 20 μL of MTT solution (5 mg/mL) was added to each well, and cells were incubated for an additional 3 h. Then the medium was carefully removed, and precipitates were dissolved in 150 μL of DMSO, shaken mechanically for 30 min, and then absorbance values at a wavelength of 570 nm were taken on a spectrophotometer (Molecular Devices, Sunnyvale, CA, USA). Values were calculated using percentage of growth versus untreated control. The IC_50_ was defined as the concentration that caused 50% inhibition of cell proliferation, and was calculated by SAS statistical software.

## 4. Conclusions

In conclusion, 36 GA derivatives possessed different antiangiogenic activities. Derivatives **4**, **32**, **35**, **36** blocked the new blood vessels from the dorsal aorta in the zebrafish embryos assay, exhibited strong inhibitory effects on the migration, tube formation and proliferation of HUVECs, additionally they showed relatively lower toxicities to zebrafish and LO2 cell line than GA. The four compounds were also capable to effectively inhibit the five tumor cell lines tested (A549, HepG2, HCT116, K562 and HeLa). Given the results of this comprehensive research, we found that compound **36** was the most potent one and may serve as a potential new antiangiogenesis candidate.
